# Gastro-tracheal fistula - unusual and life threatening complication after esophagectomy for cancer: a case report

**DOI:** 10.1186/1749-8090-4-69

**Published:** 2009-11-29

**Authors:** Jane E Nardella, Dirk Van Raemdonck, Hubert Piessevaux, Pierre Deprez, Raphaël Droissart, Jean-Pierre Staudt, David Heuker, Etienne van Vyve

**Affiliations:** 1 Department of Surgery, St Jean Hospital, Brussels, Belgium; 2 Department of Thoracic Surgery, University Hospital Gasthuisberg, Leuven, Belgium; 3 Department of Gastro-Enterology, Cliniques Universitaires St-Luc, Université Catholique Louvain, Brussels, Belgium

## Abstract

**Background:**

A gastro-tracheal fistula following esophagectomy for cancer is a rare but potentially lethal complication. We report the successful surgical closure after failed endoscopic treatment, of a gastro-tracheal fistula following esophago-gastrectomy for cancer after induction chemo-radiotherapy.

**Case presentation:**

A 58 year-old male patient presented with a distal third uT3N1 carcinoma of the esophagus. After induction chemo-radiotherapy, he underwent an esophago-gastrectomy with radical lymphadenectomy and reconstruction by gastric pull-up. Immediate postoperative outcome was uneventful. On the 15^th ^postoperative day however, our patient was readmitted in the Intensive Care Unit with severe bilateral basal pneumonia. Three days later a gastro-tracheal fistula was diagnosed upon gastroscopy and bronchoscopy. His good general condition allowed for an endoscopic primary approach which consisted in the insertion of a covered stent in the trachea along with clipping and glueing of the gastric fistular orifice. Two attempts proved unsuccessful.

**Conclusion:**

After several weeks of conservative measures, surgical re-intervention through a right thoracotomy with transection of the fistula and closure by primary interrupted sutures of both fistular orifices along with intercostal muscle flap interposition led to excellent patient outcome. Oral feeding was started and our patient was discharged.

## Background

The occurence of a gastro-tracheal fistula following esophagectomy for carcinoma is a rare(0.3-0.5% [1,2]) but potential lethal complication. Literature on this entity consists mainly of case reports. We discuss semiology, diagnostic steps and treatment options which include conservative approaches or surgical reintervention. We report a case treated in a multidisciplinary team with excellent outcome. Surgical re-intervention with fistula transection, primary suturing of gastric and tracheal defects combined with muscle-flap interposition proved successful after failed endoscopic conservative management. The importance of multimodal management within a multidisciplinary team is emphasized, especially when confronted to uncommon complex and severe surgical complications such as a gastro-tracheal fistula after esophagectomy for cancer.

## Case presentation

**Figure 1:**
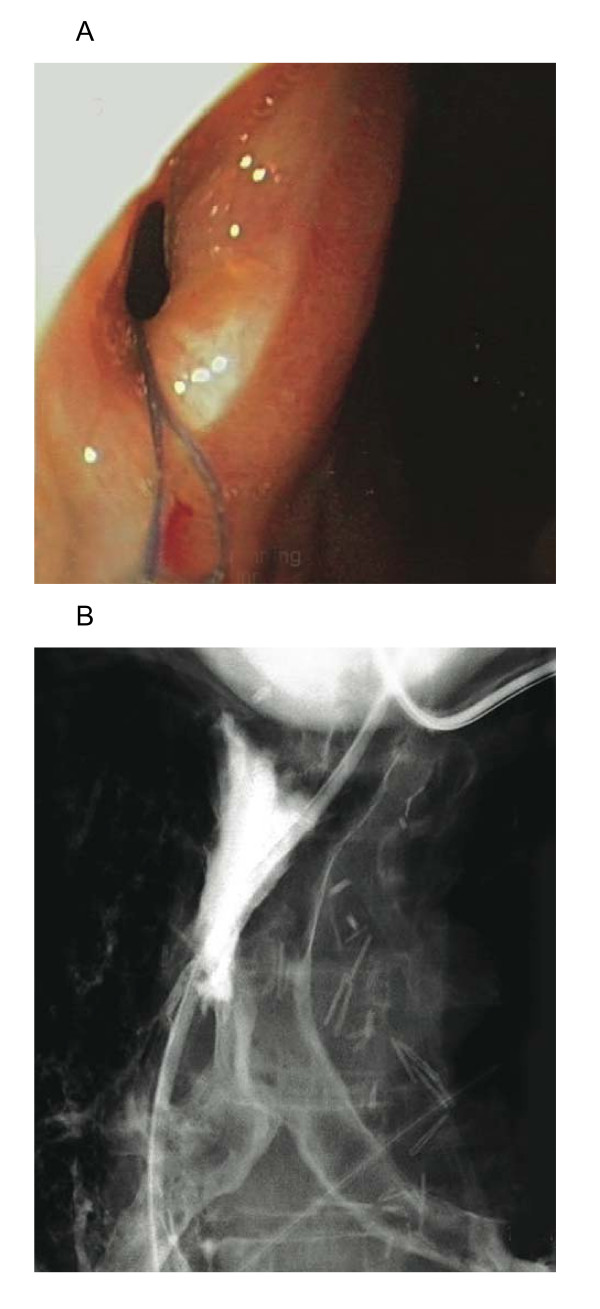
**A - Endoscopic view and diagnosis of the gastro-tracheal fistula**. Gastric. B - Barium in the tracheal bifurcation after esophagography.

A 58 year-old male patient presented with an oesophageal distal 1/3 poorly differentiated uT3N1 adenocarcinoma. He underwent induction chemotherapy (5FU, Cisplatin) and radiotherapy (45 Gy). Eight weeks later, a subtotal oesophagogastrectomy with radical lymphadenectomy and reconstruction using a gastric pull-up according to the Akiyama technique was performed. Final histopathology reported a poorly differentiated carcinoma with a total of twenty-three nodes examined, all free of disease. The immediate postoperative outcome was uneventful. On the 9^th ^postoperative day an oesophagography showed no evidence of passage disturbance. However, on the 15 th postoperative day, the patient experienced severe respiratory distress and was re-admitted to the ICU. Chest CT-Scanning showed bilateral basal pneumonia. Despite negative oesophagography on the 18^th ^postoperative day, a subsequent upper-GI endoscopy carried out 3 days later, revealed an 8 mm anastomotic orifice (fig 1a). A further esophagography (fig 1b) showed pathognomonic tracheo-bronchial opacification with contrast swallowing. Bronchoscopy showed a necrotical defect 5 cm above the carina. On the basis of these observations an anastomotic leak with a tracheo-gastric fistula was diagnosed.

**Figure 2:**
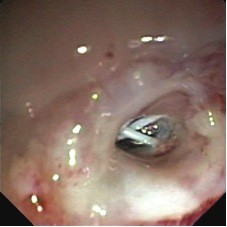
**Second endoscopic approach (endoscopic view): at this point, a shortened oesophageal self-expanding plastic stent (Polyflex, Boston Scientific) has been inserted upside down in the large diameter trachea**. The digestive end of the fistula is closed using a folded collagen mesh (Cook), Fibrin glue sealing and approximation of the walls with intraparietal cyanoacrylate injection.

**Figure 3:**
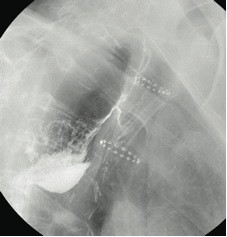
**Recurrence: esophagography with posterior extravasation of contrast barium**.

**Figure 4:**
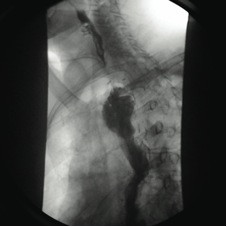
**Seventh post-operative day after "redo" thoracotomy; esophagography showing no leakage of contrast**.

The patient remained in good general condition with no signs of mediastinitis. After a multidisciplinary consultation, a decision was taken to attempt endoscopic closure of the fistula. The gastric tube didn't allow for endoscopic placement of a prosthesis to seal off the fistula because of its very large caliber. The initial approach therefore associated abrasion of the fistular orifice using Argon Plasma coagulation, approximation with endoscopic clips and fibrin glue injection. The subsequent oesophagography, unfortunately showed the persistence of the fistula. The next attempt performed 1 week later combined closure of the tracheal orifice and closure of the anastomotic leak. A shortened oesophageal self-expanding plastic stent (Polyflex, Boston Scientific) was inserted upside down in the large diameter trachea. The digestive end of the fistula was closed using a folded collagen mesh (Cook), Fibrin glue sealing and approximation of the walls with intraparietal cyanoacrylate injection (fig 2). The radiological control performed 4 days later showed closure of the fistula. Unfortunately, 2 weeks later a recurrence occurred (fig 3). After three months of conservative measures, a decision was taken for surgical re-intervention and closure of the remaining fistula. We carried out a right thoracotomy. Exploration showed a single fibrotic band creating a substenosis of the mid-gastric tube with consequent dilation of the upper gastric pouch. The gastric tube was therefore freed from fibrosis and mobilised. The fistula was exposed and transected. The gastric and tracheal defects were closed with interrupted sutures and an intercostal muscle bundle was interposed to protect the suture lines. Complete closure of the fistula was proven on contrast swallow one week later (fig 4). Oral feeding was resumed and the patient was discharged after being in hospital for 4 months.

Despite close anatomical relation ship between the trachea and the esophagus, a benign postoperative tracheo-gastric fistula after esophagectomy with gastric pull-up for cancer is a rare but life-threatening complication. Literature about this severe complication consists mainly of case-reports. There are various possible strategies; conservative, endoscopic or surgical but treatment of such fistulas remains challenging. The approach will depend on the patient's general condition and severity of symptoms, size and location of the fistula and managing team. Symptoms vary from mild coughing to more severe recurrent broncho-pneumonia and life-threatening mediastinitis. Diagnosis is based on radiologic contrast study and is often confirmed both by oesophagogastroscopy and bronchoscopy thus allowing precise localization and assessment of the fistula [2]. There are various identified predisposing factors: neo-adjuvant radio-chemotherapy and transthoracic en-bloc resection with extended mediastinal lymphadenectomy[1]. The underlying pathophysiology to this complication mainly consists of leakage of the anastomosis with mediastinal abscess formation and secondary fistulization to the trachea[1], tracheal ischemia after extensive dissection in the upper mediastinum[2]. Other reported causes consist of traumatic injuries to the trachea during surgical dissection, cuff-induced tracheal necrosis during prolonged endotracheal intubation, tracheal erosion by gastric staple line. Gastric erosion by tracheostomy tube has also been reported. Regarding operative technique, careful dissection of tumors with airway adherence and meticulous operative technique in performing esophago-gastric anastomosis are essential in the prevention of this complication. Technical errors such as unrepaired mucosal defect at the time of the anastomosis, impaired blood supply to the esophageal stump or gastric tube, tension on the suture line, overdistended transplanted stomach and oversight knotting, must be avoided[3].

Literature reports up to 50% overall morbidity and mortality in surgical reintervention for tracheoesophageal fistula after esophageal atresia repair in children [4]. In our case, with a patient in very good general condition, a well localized fistula with small fistular orifices and no sign of large adjacent necrosis, an endoscopic approach was our first management approach. We started out by ruling out the endoscopic insertion of a prosthesis in the gastric tube because of the tube's very large diameter. A first endoscopic attempt which consisted of abrasion-coagulation, fibrin glue injection, and approximation with endoscopic clips of the fistular orifice was unsuccessful. A few days later, after placing a Polyflex stent in the trachea, further fibrin glue was injected in the esophageal defect and the orifice was closed off with hemostatic clips. Use of fibrin glue, with or without hemostatic clips or Vicryl mesh in upper gastro-intestinal leaks or tracheo-esophageal fistula in children who have undergone surgery for esophageal atresia has proved successful. However, several technique factors such as early diagnosis before epithelium is formed in the fistula and clean fistula cavity are a prerequisite to the success of this procedure[5,6]. In our case, we believe failure of the fistula to close spontaneously after endoscopic management was related to the H-type direct fistular connection along with narrowing of the gastric tube with proximal dilation due to local fibrosis as was demonstrated at reoperation.

When conservative measures fail and if the patient's condition worsens, a surgical approach is necessary. Several techniques have been described most of them in the treatment of tracheo-esophageal fistulas caused by cuffed tracheal tube[7]. In these cases, surgical treatment consists of a direct approach with dissection of the fistula and closure of the tracheal and oesophageal defects[8]. Additionally, some authors have described the use of interposed vital tissue(pleural, pericardial, muscle-flap, myocutaneous flaps). These tissues have rich blood supply, they fill in dead space, protect the tracheal and gastro-esophageal suture lines and may help prevent recurrent fistulization[2,8-10].

After 3 months of conservative measures, failed endoscopic treatment and no sign of progression, our remaining option was a surgical approach. We freed the gastric tube from fibrosis through a right thoracotomy. Both the gastric tube and esophageal stump proved well-vascularized and viable thus avoiding extensive heavy excisions and bypass replacement [2]. We carried out selective dissection and transection of the fistula. The tracheal stent was removed through the fistular tracheotomy orifice. Both gastric and tracheal orifices were then closed off with interrupted sutures. We protected the repaired defects and suture lines with an interposed pedicled intercostal muscle bundle consistent with previous literature reports[2,8-10]. Contrast swallow 7 days postoperatively showed complete closure of the fistula. At 7 weeks after reintervention the patient suffered severe fatigue but was progressing steadily without any respiratory or swallowing impairment.

## Conclusion

A tracheo-gastric fistula after esophagectomy for cancer is a rare but serious and challenging complication. Multimodal management with a first conservative endoscopic approach proved unsuccessful. Even though we had to perform a "redo" thoracotomy in a previously irradiated field, closure of the fistula along with pedicled intercostal muscle bundle interposition allowed excellent outcome. We consider this approach, feasible and safe in the management of failed conservative approach of a tracheo-gastric fistula following esophagectomy.

## Consent

Written informed consent was obtained from the patient for publication of this case report and any accompanying images.

## Competing interests

The authors declare that they have no competing interests.

## Authors' contributions

JN: Intern in cardiothoracic surgery, main author

DVR: Thoracic surgeon, performed the redo thoracotomy, revised critically the draft

HP: Gastroenterologist, performed the conservative endoscopic treatment, revised the draft

PD: Gastroenterologist, performed the conservative endoscopic treatment, revised the draft

RD: Visceral surgeon, provided advice during the writing process

JPS: Thoracic and visceral surgeon, provided advice during the writing process

DH: Visceral surgeon, provided advice during the writing process

EVV: Thoracic and visceral surgeon, performed the esophagectomy, coached the research and writing process, revisited the draft
